# Epigenetic alterations in preneoplastic and neoplastic lesions of the cervix

**DOI:** 10.1186/1868-7083-4-13

**Published:** 2012-08-31

**Authors:** Kathleen P Saavedra, Priscilla M Brebi, Juan Carlos S Roa

**Affiliations:** 1 Universidad de La Frontera, School of Medicine, Department of Pathology, Molecular Pathology Laboratory, BIOREN-CEGIN, Temuco, Chile; 2 Pontificia Universidad Católica de Chile, School of Medicine, Department of Pathology, Santiago, Chile; 3 Department of Pathology, School of Medicine, Universidad de La Frontera, Manuel Montt 112 of 211, Temuco, Chile

**Keywords:** Cervical cancer, Human papillomavirus, Epigenetics alterations, Biomarkers

## Abstract

Cervical cancer (CC) is one of the most malignant tumors and the second or third most common type of cancer in women worldwide. The association between human papillomavirus (HPV) and CC is widely known and accepted (99.7% of cases). At present, the pathogenesis mechanisms of CC are not entirely clear. It has been shown that inactivation of tumor suppressor genes and activation of oncogenes play a significant role in carcinogenesis, caused by the genetic and epigenetic alterations. In the past, it was generally thought that genetic mutation was a key event of tumor pathogenesis, especially somatic mutation of tumor suppressor genes. With deeper understanding of tumors in recent years, increasing evidence has shown that epigenetic silencing of those genes, as a result of aberrant hypermethylation of CpG islands in promoters and histone modification, is essential to carcinogenesis and metastasis. The term epigenetics refers to heritable changes in gene expression caused by regulation mechanisms, other than changes in DNA sequence. Specific epigenetic processes include DNA methylation, chromotin remodeling, histone modification, and microRNA regulations. These alterations, in combination or individually, make it possible to establish the methylation profiles, histone modification maps, and expression profiles characteristic of this pathology, which become useful tools for screening, early detection, or prognostic markers in cervical cancer. This paper reviews recent epigenetics research progress in the CC study, and tries to depict the relationships between CC and DNA methylation, histone modification, as well as microRNA regulations.

## Review

### Cervical cancer

Cervical cancer (CC) is the second most prevalent neoplasia in women worldwide and the fifth cause of death by cancer in this population, posing a significant public health problem [1-3]. The incidence of CC and its precursor stages is high mainly in developing countries [4]. In 2008 an incidence of 15.8 and a mortality of 8.2 for every 100,000 women was estimated. Around 529,000 new cases are detected every year, with nearly half of these cases dying [5].

The pathogenesis of CC begins as a slow process that interrupts the normal differentiation of the cervical squamous epithelium, thereby producing changes in its structure and physiology [6]. It initially presents through precursory lesions that evolve slowly and progressively, which can then advance to slight, moderate, and severe stages of dysplasia. CC may evolve into cancer *in situ*, which is limited to the epithelial surface, and/or into invasive cancer, in which case the involvement goes beyond the basement membrane [7]. Therefore, one of the characteristic factors of CC is its defined clinical stages, which are associated with the different evolutionary stages that lead to the development of the carcinogenesis [8].

It has been firmly established, both biologically and epidemiologically, that the main cause of CC is due to a persistent infection of high-risk human papillomavirus (HPV) types, which are present in 99.7% of CC cases [9]. Nevertheless, the presence of a persistent high-risk HPV infection risk is not sufficient to immortalize and transform the epithelial cells of the host; it has been confirmed that the presence of genetic and epigenetic alterations are needed for the development of carcinogenesis. As a result, these factors taken together may alter the control of the cell cycle, causing the host cell to acquire an immortal phenotype and ultimately progress towards a malignant and invasive phenotype [10].

### Human papillomavirus

HPV is a small, non-enveloped virus belonging to the Papillomaviridae family of viruses [11]. It contains a circle of double-stranded DNA approximately 8 kb in length [9,12,13]. Generally, the HPV genome is composed of three basic regions: an early region, composed of six open reading frames (ORFs) known as E1, E2, E4, E5, E6, and E7; a delayed region, with two coding ORFs for the structural viral proteins L1 and L2; and an upstream regulatory region (URR) [14].

**Figure 1:**
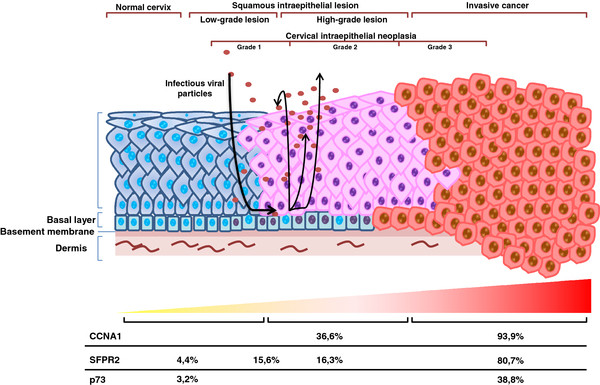
**Progression of cervical cancer by HPV and promoter methylation during progression.** Adapted from Woodman *et al*., 2007 [5]. The HPV virus has access to the basal cells by means of microlesions present in the cervical tissue. The virus is maintained in episomic form with a low number of copies (cells with a purple nucleus). The cells of the basal layer are divided and begin to migrate towards the surface. The untreated lesions progress to microinvasive and invasive cancer, where the HPV genome is associated with the host genome (cells with red nuclei). In the superficial layer the viral DNA is packed in capsids and the progeny is released to reinitiate the infection.

The HPV life cycle depends on the replication machinery of the host cell and the differentiation of the squamous epithelium of the cervix [9,15]. The cycle begins when infectious viral particles arrive at the basal layer of the epithelium, where they enter the host cell. In the cells of the basal layer of the epithelium, the virus remains stable within an episome with a low number of copies, making up the virus reservoir [15,16]. Once the infected cells of the basal layer are divided, some migrate towards the superficial layer, lose their ability to divide, and initiate their terminal differentiation, whereas the others remain in the basal layer, self-renewing the population and maintaining the infection. In the external layers of the epithelium, the viral DNA is packed in capsids and the lineage is released to initiate the infection again (Figure 1) [9,10,15,16].

During the progression of the carcinogenesis, the HPV genome is integrated into the genome of the host cell, so that the virus not only has a complete life cycle but also manages to persist in the host cell. During this integration the coding regions for E1 and E2 are usually interrupted, resulting in the loss of expression from the viral E2 gene, which acts as a repressor of the gene expression of E6 and E7, thus allowing an increase in the expression of both proteins. The form by which E6 and E7 act includes the inactivation of p53 and the hypophosphorylated retinoblastoma protein (pRB), two classic tumor suppressor genes [17]. Currently it is known that E6 and E7 are also linked to various proteins involved in the processes of cell adhesion, including but not limited to apoptosis, cell cycle, DNA repair, metabolism, translation, and transcription signals [6,12,14,17-19].

### Epigenetics in CC

Epigenetics is defined as all those heritable changes in gene expression that cannot be explained by an alteration in the DNA nucleotide sequence [10]. The epigenetic modifications most studied are DNA methylation and histone modification. Recently a new [10] epigenetic phenomenon has been suggested, microRNA (miRNA), which has been implicated in transcriptional silencing [20,21].

A series of epigenetic alterations have been identified that occur during each of the stages of CC, both in the HPV and in the genome of the host cell [10]. One of the earliest pieces of evidence on record with respect to this subject was thanks to a [3 H]-S-adenosylmethionine incorporation assay performed on cervical neoplastic tissue. In this study, it was observed that the degree of hypomethylation (degree of incorporation of the [3 H]-methyl group) increased progressively together with the advance of the cervical neoplasia [22]. This finding was confirmed by later studies that involved quantitative analyses of DNA methylation supported by computer analysis on interphase nuclei by immune-labeling with anti-5-methylcytosine antibodies. A progressive demethylation of the tissue was observed in samples of dysplasia and cancer compared to normal controls [23,24].

Indeed, most tumor suppressor genes possess promoter regions rich in CpG islands, which tend to be hypermethylated in CC, leading to the inactivity of these genes [20].

Identification of numerous epigenetic alterations in all cervical carcinogenesis stages reiterates the potential value of methylation markers for improved diagnosis. Twelve studies have been identified methylation markers in numerous specimens (serum/plasma, liquid-based cytology, cytobrush samples, and urine) of cervical cancer patients. The best performing panel of genes (methylation status for four genes: CALCA, DAPK, ESR1, and APC) was discovered by Wisman *et al.*, in cervical scrapings. Using this mathylation panel a sensitivity of 89% and a specificity of 100% was achieved in order to identify cervical cancer patients [25,26].

### Methylation in CC

There are numerous reports demonstrating that abnormal promoter hypermethylation leads to silencing or diminished expression of tumor suppressor genes in cervical carcinoma. Epigenetic silencing due to the methylation of genes involved in different regulatory pathways, such as apoptosis, cell cycle control, and DNA repair, among others, has been widely investigated. What follows is a brief review of the genes described as methylated in cervical carcinogenesis according to the pathway involved [4].

#### Cell cycle

It is well established that cancer cells evolve as a result of a deregulation in the normal cell cycle. The adequate regulation of the cell cycle is essential for all cell types and requires a great number of participant molecules, such as cyclin-dependent kinases, along with other natural inhibitors like p16, Rb, and p15, which play an essential role [27]. For its part, high-risk HPV interferes with the control of the normal cell cycle by means of its viral E6 and E7 oncogenes, which have the ability to bind to the regulatory proteins of the host cell, particularly to tumor suppressor genes like p53 and pRB. This event might cause the degradation of p53 on the part of E6 and the inactivation of pRB by binding it to E7 [28], which in turn permits the overexpression of the cyclin-dependent kinase inhibitor p16 (p16^INK4A^), leading to genetic instability [29]. p16^INK4A^ (MTS1, CDKN2) encodes a nuclear phosphoprotein involved in the negative regulation of the cell cycle through the inhibition of CDK4 or −6 by binding to the cyclin D1, which regulates the G1 phase of the cell cycle. This mechanism enables the deregulation of pRB1 at the checkpoint of the G1/S phase, thereby affecting cell proliferation [29,30]. Accordingly, cases have been reported in which the p16^INK4A^ protein was found to be strongly expressed in dysplasias and malignant cells of the squamous and glandular epithelium of the cervix [31-33]. It is argued that due to the action of the high-risk HPV viral oncogene E7 on the Rb protein, a negative feedback is produced that leads to the overexpression of p16^INK4A^[34,35]. However, the expression of p16^INK4A^ in CC not associated with infection by HPV might be explained by independent pathways of the virus [33]. It has been shown that the inactivation of the tumor suppressor gene p16^INK4A^ might be mediated by different genetic and epigenetic mechanisms, the latter of which include hypermethylation of the CpG islands in gene promoter regions; nevertheless, this event is present only infrequently in cases of CC associated with infection by HPV. In these reported cases, it has been observed that the degree of methylation increases as the carcinogenesis progresses [10,30,33,36], being translated in the expression silencing of p16^INK4A^. This in turn may provoke the binding of the different CDKs involved in the cyclin D1 pathway, leading to the phosphorylation of Rb, this being a key mechanism for releasing the cell cycle from control by Rb [14].

Cyclin A1 (CCNA1) is a cell cycle regulator essential at the start of metaphase in male meiosis. Thus, a high expression of CCNA1 has been found in testicular cells and hematopoietic progenitors; nevertheless, its expression is low in many other types of cells [37,38]. Kitkumthorn *et al*. showed that the CCNA1 promoter region is methylated in cases of CC, and the degree of methylation advances as the oncogenesis progresses. Therefore, in normal samples and in low-grade lesions, no methylation was found; whereas in high-grade lesions, microinvasive cancer, and invasive cancer, 36.6%, 60%, and 93.3%, respectively, were found.

#### DNA repair

Silencing of the DNA repair gene *O*^*6*^*-methylguanine-DNA methyltransferase* (*MGMT*) by methylation of its promoter region is an early event that occurs in various cancers. This gene encodes a DNA repair protein that transfers and accepts alkali groups from the O^6^ position of the guanine, thereby preventing *G > A* mutations in the genome [39]. Iliopoulos *et al.* observed that methylation of this gene increases progressively as the stages of CC advance [40]; similar results were obtained by Lin *et al*. [33].

In addition to being associated with control of the cell cycle, the *CCNA1* gene is also involved in the repair of double-strand DNA breaks affected by radiation [41]. Hypermethylation of the *CCNA1* promoter has been associated with a loss of p53 function, which has generally been associated with a degradation resulting from viral oncoprotein E6 [42].

Other genes less frequently related to DNA repair and silenced by methylation of their promoter regions are *hMLH1* and *FANC*[10,43].

#### Cell differentiation and proliferation

The Wnt family of proteins involves a wide variety of growth factors that regulate cell differentiation, proliferation, migration, and oncogenesis during embryonic development [44]. It has been shown that the aberrant activation of the Wnt signaling pathway takes place during carcinogenesis, inhibiting the apoptosis of tumor cells in different human cancers. When Wnt ligands are present, they bind to Frizzled (Fz) transmembrane receptors*,* activating the dishevelled cytoplasmatic protein by phosphorylation. The activation of this protein leads to the activation of the Wnt signaling cascade that culminates in the accumulation of β-catenin in the cytoplasm. This is deposited in the nucleus, where it forms complexes with members of the TCF/LEF cells and increases the transcription of TCF/LEF-dependent target genes, such as *c-myc*, *cyclin D1*, and *TCF-1*. In normal conditions, that is, in the absence of the carcinogenic process, the family of secreted frizzled-related proteins (SFRPs) is the one responsible for antagonizing the Wnt signaling pathway, among other antagonists. SFRPs bind directly to the Wnt proteins and block their interaction with the Fz transmembrane receptors. As the Wnt signaling pathway is blocked, the intracellular β-catenin is phosphorylated, ubiquitinated, and finally degraded by proteosomes. Thus, the absence in the nucleus of β-catenin and TCF/LEF interaction might repress oncogene transcription [45-47]. It has been suggested that the epigenetic silencing of antagonists of the Wnt signaling pathway might result in the aberrant activation of this pathway, leading to carcinogenesis.

#### Apoptosis

The apoptosis pathway is interrupted in most human cancers by various phenomena that affect the pathway’s main components, as for example the suppression of p14ARF, overexpression of MDM2, or mutation of p53 [48,49].

The p53 pathway responds to stress signals, as they are the interruption in DNA replication fidelity and cell division. These signals activate the p53 pathway specifically by means of post-transcriptional modifications, enabling the arrest of the cell cycle, induction of senescence or cell apoptosis [48,49]. One of the protein layers to induce apoptosis is p73, a member of the p53 family of tumor suppressor proteins, a protein with both a strong structural as well as functional homology.

This leads to this protein, when overexpressed, possibly activating the transcription of p53-responsive genes, such as *p21*, *Bax*, *MDM2*, and *GADD45*, and to cell growth inhibition by apoptosis induction [49,50]. P73 transcription is regulated by promoter regions and the first exon of gene *p73*, regions rich in CpG dinucleotides. Methylation in the cytokine residues of CpG dinucleotides present in these regions plays a key role in the regulation of *p73* gene expression due to epigenetic modifications. The transcriptional silencing of *p73* has been demonstrated in leukemia, lymphomas, brain tumors, and ovary cell lines [51,52]. Recent studies have reported the existence of epigenetic modifications that might act on *p73* through the hypermethylation of their CpG islets, making it an important inactivation mechanism of the gene expression of *p73* in CC. In this study it was observed that 38.8% of samples with CC presented hypermethylation of the *p73* gene *vs.* 3.2% in control samples. The hypermethylation of *p73* was significantly related to the reduction or suppression of *p73* expression. It is important to emphasize that the expression of *p73* is associated with favorable responses to radiation therapy in CC, whereas suppression of the protein is related to radioresistance [53].

### Histone modification

Cancer cells show histone modification patterns in individual genes and generally at the nuclear level in individual cells.

Histone acetyl transferases (HAT) and deacetylases (HDAC) are two enzymes with opposite activities, responsible for regulating the transcriptional machinery, controlling the state of histone acetylation. It has been shown that these enzymes might be involved in cell proliferation, cell differentiation, and regulation of the cell cycle [54-56]. As a result, deregulation of the state of histone acetylation at cell level might be related to the carcinogenic process. In CC it has been shown that HDAC1 and 2 are overexpressed in cases of dysplasia and CC. The relation that presents the suppression of HDAC2 with the increase of apoptosis, associated with an increase in the p53-independent expression of p21^Cip1/WAF1^ was also determined [54].

The *MGMT* gene, the participation of which in DNA repair was detailed previously, is silenced in cases of CC by histone deacetylase action [57]. It has also been reported that the acetylated and phosphorylated forms of histone H3 in cytological smears display a noticeable association of histone H3 modification with the progression of carcinogenesis from low-grade to high-grade lesions [58].

It has recently been demonstrated that the therapeutic use of histone deacetylase inhibitors in CC has a high anti-cancer potential [59].

### miRNA

Several studies have proven the existence of miRNA expression profiles in cervical cancer*.* Lee *et al.* described the overexpression of 10 miRNA in CC: miR-199-s, miR-9, miR-199a*, miR-199a, miR-199b, miR-145, miR-133a, miR-133b, miR-214, and miR-127, and only two repressed, miR-149 and miR-203 [60]. More recently Pereira *et al.* demonstrated that a certain variability exists with respect to the miRNA expression profiles; they described eight miRNA that show a relative reduction in expression during the development of CC, from normal tissue to atypical dysplasia and cancer: miR-26a, miR-143, miR-145, miR-99a, miR-203, miR-513, miR-29a, and miR-199a. Nevertheless, they described another six miRNA that showed a drop in expression from the transition from normal tissue to premalignant dysplasia, but that returned to normal levels with the onset of cancer: miR-106a, miR-205, miR-197, miR-16, miR-27a, and miR-142-5p [61].

miR-21 has been described as an oncogene in six types of cancer; in CC it may promote cell proliferation and might repress the expression of programmed cell death 4 (PCD4) [62]. The expression of miR-218 is reduced in CC, its transcriptional target is laminin-5 β3 (LAMB3), the expression of which increases in cases of CC, and this has been reported as a marker of invasiveness in cervical lesions (Table 1) [63].

**Table 1:** miRNA expression in cervical cancer

**miRNA**	**State**	**References**
miR-199-s	Overexpressed	[60]
miR-9	Overexpressed	[60]
miR-199a*	Overexpressed	[60]
miR-199a	?	[66- [61]
miR-199b	Overexpressed	[60]
miR-145	?	[66- [61]
miR-133a	Overexpressed	[60]
miR-133b	Overexpressed	[60]
miR-214	Overexpressed	[60]
miR-127	Overexpressed	[60]
miR-149	Repressed	[60]
miR-203	Repressed	[61]
miR-26a	Repressed	[61]
miR-143	Repressed	[61]
miR-99a	Repressed	[61]
miR-513	Repressed	[61]
miR-29a,	Repressed	[61]
miR-106a	Repression and subsequent normalization	[61]
miR-205	Repression and subsequent normalization	[61]
miR-197	Repression and subsequent normalization	[61]
miR-16	Repression and subsequent normalization	[61]
miR-27a	Repression and subsequent normalization	[61]
miR-142-5p	Repression and subsequent normalization	[61]
miR-21	Overexpressed	[62]
miR-218	Repressed	[63]

Thereby, the relationship between cervical carcinogenesis and epigenetics alterations like DNA methylation, histone modifications, and miRNAs have received increasing attention for their potential involvement in the development of CC, especially its usefulness as biomarkers of carcinogenesis. Epigenetic alterations can serve as biomarkers in clinical studies and could be in medical diagnostic. The reversible nature of epigenetic alterations can be considered also for therapeutic approaches of advanced stage cervical cancer.

## Conclusions

In spite of being a relatively new area of study, epigenetic alterations have enabled the understanding of the differences present in the gene expression profiles in different diseases, including carcinogenic processes. This way, these alterations have become a powerful line of investigation for the establishment and progression of cancer. As far as CC is concerned, we can highlight the ample number of genes affected by epigenetic alterations at the level of gene methylation, histone modification, and miRNA action. The emergence of this recent information, valuable for the area of clinical diagnosis, has made it possible to establish candidate genes that are useful for the search of early detection biomarkers. Thus the establishment of methylation profiles, histone modification maps, and miRNA expression profiles has become the aim of many investigators, so as to be able to propose new more sensitive and specific alternatives for CC screening. In the same way, these epigenetic alteration profiles could be used for the prognosis of the disease, for the assessment of the patient’s evolution prior to the administration of a certain therapy and for its implementation.

## Abbreviations

CC: Cervical cancer; CCNA1: Cyclin A1; DNTMs: DNA methyltransferase; DKK-1: DICKKOPF-1; FHIT: Fragile histidine triad; Fz: Frizzled; HAT: Histone acetyl transferases; HDAC: Histone deacetylases transferases; pRB: Hypophosphorylated retinoblastoma protein; HPV: Human Papilloma Virus; LAMB3: Laminin-5 β3; MGMT: O^6^-methylguanine-DNA methyltransferase; miRNA: MicroRNA; ORFs: Open reading frames; SFRPs: Secreted frizzled-related proteins; URR: Upstream regulatory region.

## Competing interests

The authors declare that they have no conflicts of interest.

## Authors’ contributions

KS designed the article and participated in writing of the manuscript. PB revised the manuscript and contributed to discussion. JCR provided guidance for the overall structure and content of the manuscript. All authors read and approved the final manuscript.
